# Angiopoietin-like Proteins in Colorectal Cancer—A Literature Review

**DOI:** 10.3390/ijms22168439

**Published:** 2021-08-05

**Authors:** Monika Zajkowska, Barbara Mroczko

**Affiliations:** 1 Department of Neurodegeneration Diagnostics, Medical University of Bialystok, 15-269 Bialystok, Poland; mroczko@umb.edu.pl; 2 Department of Biochemical Diagnostics, Medical University of Bialystok, 15-269 Bialystok, Poland

**Keywords:** ANG, ANGPTL, colorectal cancer, tumor markers, gastrointestinal tract tumors, angiogenesis

## Abstract

Colorectal cancer (CRC) is one of the most common types of malignancy, with an annual incidence of about 10% of the total number of new cases. Despite well-developed screening tests, mortality from this type of cancer remains unchanged. Therefore, it is important to search for more accurate markers that are useful in the detection of colorectal cancer (especially in its early stages), and treatment. Angiopoietin-like proteins (ANGPTLs) are a family of eight proteins with a diversity of applications, including pro- and anti-angiogenic properties. Consequently, we performed an extensive search of the literature, pertaining to our investigation, via the MEDLINE/PubMed database. Based on the available literature, we summarize that some of those proteins are characterized by increased or decreased concentrations during the course of CRC. We can also assume that some ANGPTLs can inhibit the development of CRC, while others induce its progress. Moreover, some factors are dependent on the stage or histological type of the tumor, the presence of hypoxia, or metastases. Most importantly, some ANGPTLs can be useful in anti-cancer therapy. Therefore, further studies on ANGPTLs as potential markers of CRC should be continued.

## 1. Colorectal Cancer

Despite the progress made in the field of prophylaxis, in regard to diagnosis and treatment of malignant neoplasms, treatment outcomes are still unsatisfactory. Preventing and treating malignant tumors is a key public health concern, and a critical challenge to the healthcare system. Neoplastic diseases are some of the most persistent challenges in modern medicine. Approximately 19.3 million new cancer cases and nearly 10 million deaths were detected alone in 2020. Colorectal cancer (CRC) is one of the most common malignancies. According to the World Health Organization (WHO), CRC comprises about 10% and 9.4% of all cases, respectively [1]. Currently, colorectal cancer ranks third in terms of detection in women and second in mortality, and third in terms of both detectability and mortality in men. This type of cancer is highly associated with socioeconomic development. In highly developed and rapidly developing countries, the incidence of CRC is much more frequent than in underdeveloped countries. It is mainly associated with low physical activity, a lot of stress, alcohol abuse, environmental pollution, smoking, diet (high meat consumption, especially red meat), and obesity [1,2]. However, societal awareness of CRC is increasing every year, particularly in highly developed countries. Primary prevention includes the dissemination of epidemiological knowledge relating to the causes of cancer incidence and promotion of pro-health behaviors. Larger amounts of fiber, whole grains, and dairy products should be introduced into the diet, as they significantly improve the functioning of the intestines and reduce the risk of malignant changes [1,3,4]. Several studies have reported that supplementing with calcium or vitamin D3 may have anti-disease effects [2,3]. In addition, it has been proven that chronic use of acetylsalicylic acid and other nonsteroidal anti-inflammatory drugs reduces the risk of CRC [4]. Not smoking (or quitting smoking) also reduces the risk of developing CRC and many other tumors [5].

Prevention is one of the best methods of fighting cancer. Primary prophylaxis is directed at preventing malignant neoplasms through all activities aimed at reducing the risk of developing cancer. Primary prophylaxis may reduce mortality and the incidence of malignant neoplasms by determining the factors influencing the development of the disease, understanding its mechanisms of action, and promoting pro-health behaviors. Apart from primary prevention, secondary prevention (screening) plays a significant role. Screening tests enable the detection of precancerous conditions or an early form of malignant neoplasm. Early cancer detection could enhance a patient’s chances of successful treatment and reduce the risk of metastasis or recurrence, extending the patient’s length of life and improving the patient’s comfort level. Moreover, we observed that there is an increasing number of screening programs and a development in screening tests. We also observed an increasing number of patients applying for these programs, not only those at risk of developing CRC, but also without risk factors [1].

Currently, as a part of preventive/screening tests of benign lesions (adenomas) and colorectal cancer, tests are available, including the fecal occult blood test (FOBT), fecal immunochemical test (FIT), colonoscopy, sigmoidoscopy, colonography, or a multi-target stool deoxyribonucleic acid (mt-sDNA) test [6]. FOBT, and its more advanced version, FIT, should be performed every 12 months. These diagnostic methods allow detecting the presence of blood (hemoglobin peroxidase), which does not cause macroscopic changes in the appearance of the stool. Most tests do not even require special preparation (as was the case earlier with the guaiacol method used in FOBT), such as the consumption of meat (especially red meat), iron supplementation, or green vegetables, as these test are based on specific antibodies against the human hemoglobin. These types of tests are the most commonly used due to their high availability and non-invasiveness. Nevertheless, there are some weaknesses with these tests, including quite low sensitivity and inability to detect pre-cancerous changes. A positive result of FOBT or FIT is an indication for colonoscopy. Other screening tests are sigmoidoscopy and colonography, which should be implemented every 5 years. Both of those methods are described as highly sensitive, but they also have limitations (i.e., unpleasant bowel preparation, high costs of test performance). The highest sensitivity can be acquired via a colonoscopy, which is the most common invasive method. It should be conducted every 10 years, but there is a risk of bowel perforation or bleeding. A multi-target stool DNA (mt-sDNA) test is not frequently used, but it shows sensitivity comparable to colonoscopy, sigmoidoscopy, and colonography [6,7].

In CRC, diagnostics laboratory tests can also be used. Tumor markers are specific substances, mainly proteins, which are produced by cancer cells or by healthy cells in response to the appearance of malignancy. Many tumor cells produce antigens, which are substances that may indicate the development of cancer. Some antigens are produced by both healthy and cancerous cells, but other abnormalities can be observed, i.e., the amount of secreted substance into the bloodstream in cancer development. Markers, such as carcinoembryonic antigen (CEA) or carbohydrate antigen 19-9 (CA 19-9) are routinely checked in patients with colorectal cancer. Nonetheless, tumor markers used in laboratory diagnostics, due to their low sensitivity and diagnostic specificity, are not used to detect neoplastic changes (screening tests), they are mainly used to help in monitoring of treatment or detection of tumor recurrence after surgery. Therefore, it is vital to search for new, more accurate markers with high diagnostic sensitivity and specificity in the detection of colorectal cancer, in the early stages of the disease [8].

## 2. Angiopoietin-like Proteins

**Figure 1:**
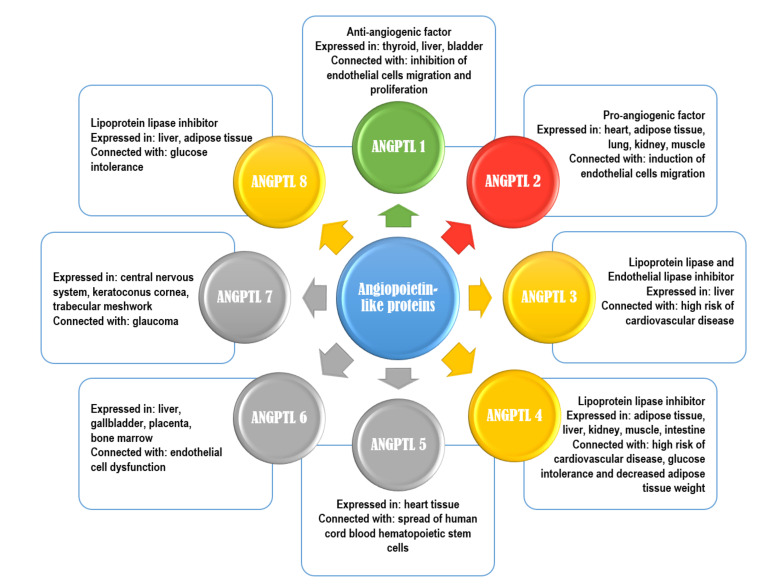
Characteristics of angiopoietin-like proteins. Green color-anti-angiogenic properties; red color-pro-angiogenic properties; yellow color-inhibitor of lipoprotein lipase; grey color-no specific function.

**Figure 2:**
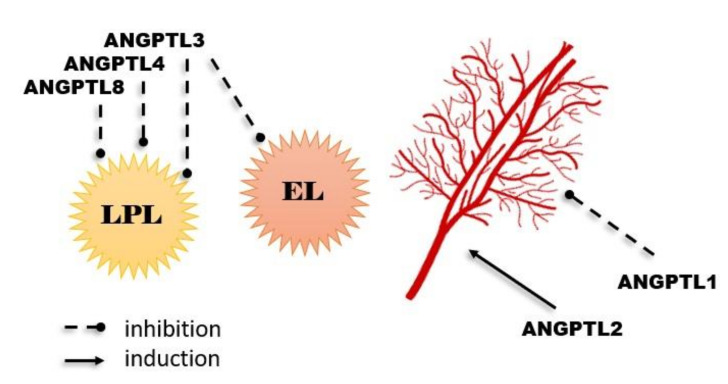
Most important physiological functions of angiopoietin-like proteins.

Angiopoietin-like proteins (ANGPTLs) are a family of proteins that are structurally similar to angiopoietins (ANG) [9]. So far, eight ANGPTLs have been discovered (they are numbered from 1 to 8) [10]. Characteristics of ANGPTLs are shown in Figure 1. Seven of ANGPTLs (ANGPTLs 1 to 7) exhibit an N-terminal coiled-coil domain and a C-terminal fibrinogen-like domain. Only one angiopoietin-like protein, ANGPTL8, which is homologue of the ANGPTL3 N-terminal domain, lacks a C-terminal fibrinogen-like domain. Their structures are remarkably similar to that of angiopoietins, as ANGs have two of the same domains as ANGPTLs 1 to 7, an N-terminal coiled-coil domain that mediates homo-oligomerization and a C-terminal fibrinogen-like domain that binds the endothelial-specific receptor tyrosine kinase (TEK) [11,12,13]. Angiopoietin-like proteins are proteins that, in humans, are encoded by genes localized in chromosome 1 (ANGPTLs 1, 3, and 7), chromosome 9 (ANGPTL2), chromosome 11 (ANGPTL5), and chromosome 19 (ANGPTLs 4, 6, 8) [12]. They have been also considered orphan ligands as they do not bind to the receptors that normally bind angiopoietins (i.e., tyrosine kinase with immunoglobulin-like and EGF-like domain 1 (Tie1), and the previously mentioned endothelial-specific receptor tyrosine kinase (TEK or Tie2)). This may be the main difference between ANG and ANGPTL, as it indicates that the mechanism of the ANGPTL action may be dissimilar from that of ANG. However, numerous studies show that ANGPTLs, similar to ANGs, are able to effectively intensify or regulate the metabolism of lipids and glucose, as well as angiogenesis (Figure 2) [9,10,11,12,13].

For example, ANGPTLs 3, 4, and 8 have the possibility of inhibiting the activity of lipoprotein lipase (LPL), which is predominantly responsible for triacylglycerol (TG) clearance in blood plasma. As high TG levels are an important risk factor for cardiovascular disease, researchers checked for reduced plasma TG levels in humans with loss-of-function alleles of ANGPTLs 3 or 4. The described “dependence” was noticed, and a reduced risk of cardiovascular disease was observed. Additionally, overexpression of ANGPTL4 (which is expressed and synthesized in adipose tissue, liver, kidneys, muscles, heart, brain, thyroid, and intestines) can intensify the intracellular lipolysis of TG inside adipocytes, which results in decreased adipose tissue weight [14,15,16,17,18]. In addition, ANGPTL3 (similar to ANGPTL8, practically entirely expressed in the liver and in a small part of the kidney and adipose tissue) is considered an inhibitor of endothelial lipase (EL). EL occurs on endothelial cells and has a part in HDL catabolism, primarily having a role in the hydrolysis of phospholipids transported by high-density lipoproteins (HDL). Moreover, ANGPTL3 loss-of-function mutations are connected to familial combined hypolipidemia, as its deficiency is correlated with reduced cholesterol and triglyceride levels in all lipoprotein fractions [19,20,21]. 

Interestingly, some authors revealed that the level of ANGPTL4 in plasma and its mRNA expression in adipose tissue can be decreased by hyperinsulinemia, which means that ANGPTL4 is involved not only in the angiogenesis process, but may also play a role in the course of diabetes mellitus or metabolic syndrome. It is believed that ANGPTL4 is also responsible for tumor development, vascular stability, glycemic homeostasis, lipid metabolism, cell differentiation, wound healing, inflammation development, and the regulation of redox potential [22]. It was also shown that reduced levels of ANGPTLs 4 and 8 might improve glucose tolerance [23,24]. Additionally, high serum levels of ANGPTL6 (expressed in the liver, gallbladder, placenta, and bone marrow) have been found in patients with metabolic syndrome and in women with subsequent pregnancy-induced hypertension. That is why its potential role in endothelial dysfunction was suggested. In addition, overexpression of this protein in animals was connected with weight loss and increased insulin sensitivity even in a high-fat diet. This protein influences wound healing by improving vascularization and proliferation of epidermal cells [25]. 

Angiogenesis is a process by which new blood vessels are formed. In physiological conditions, progression of angiogenesis is controlled by numerous factors, which may have pro- or anti-angiogenic properties. The most significant stimulators of these mechanisms are proteins of the VEGF family, fibroblast growth factor (FGF) family, angiopoietin/Tie system (ANG and ANGPTL), or metalloproteinases (MMPs). Formation of the blood vessels network is not always a physiological process. When the balance between the factors inhibiting and stimulating the angiogenesis is disturbed, pathological processes take place. Moreover, uncontrolled and deregulated recurrence of angiogenesis, as a response to extended or excessive inflammation in some pathological processes, is also found as malignant tumor development and cancer progression. Intensive tumor cell proliferation requires an enhanced supply of oxygen and nutrients. In cancer cells, this process is fostered by the induction of angiogenesis-stimulating factors. Its regulation depends on growth factors, cytokines, and cellular processes triggered in response to inflammatory stimuli or ischemia. As it is well known that inflammation promotes tumorigenesis and tumor progression, most ANGPTLs may have some functions in cancer progression [13,26,27].

Some ANPTLs may have pro- or anti-angiogenic properties. For example, ANGPTL2 was reported to be a proangiogenic factor. It is mainly expressed and synthesized in the heart, adipose tissue, lung, kidney, endothelial cells, and skeletal muscle tissues, where during hypoxia, its production is raised, which induces angiogenesis and migration of endothelial cells. ANGPTL2 expression in the adipose tissue is mainly connected with the circadian rhythm, with peak concentration during the period of activity. Adipose tissue ANGPTL2 regulates adipocyte differentiation and increases insulin sensitivity. The source of this protein in the adipose tissue are macrophages, and its expression has also been shown in the kidney subocytes of patients with diabetic nephropathy [28,29]. In contrast, ANGPTL1 (angioarrestin) was reported to be an anti-angiogenic protein. It was found that ANGPTL1 is normally synthesized and expressed in the thyroid, liver, muscle, connective tissue, bladder, and gallbladder [26]. Angiopoietin-like protein 1 can also inhibit the proliferation of endothelial cells, the migration of these cells, and their adherence to fibronectin, which is related to the inhibition of the formation of new blood vessels. The presence of this protein was found in many pathological tissues, and a reduction of its expression in the corresponding neoplasms was observed. The anti-angiogenic effect of ANGPTL1 was also confirmed in the tumor cell lines of lung and breast cancer. That is why the inhibition of cancer cell growth and tumor metastasis is a major effect of ANGPTL1 [12]. 

Little is known about the other angiopoietin-like proteins. ANGPTL5 is synthesized in heart tissue and has a functional part in the spread of human cord blood hematopoietic stem cells [30]. While ANGPTL7 is synthesized and expressed mainly in the central nervous system, keratoconus cornea, and trabecular meshwork, it is believed to be involved in the pathophysiology of glaucoma. Its presence was first demonstrated in the cornea of the eye, where its function was associated with the absence of blood vessels in this area. A relationship was also demonstrated between the expression of ANGPTL7 and the co-production of extracellular matrix components, in particular type I collagen, which is important in maintaining the correct structure of the eye cornea and fluid perfusion in the trabecular meshwork tissue [31].

Proteins from the ANGPTL family, although they are produced in various tissues, i.e., in the liver, adipose tissue, brain, and many others, they are also present in the blood. Many of them were shown to correlate with biochemical parameters used in the diagnosis and monitoring of metabolic diseases, including type II diabetes. Testing the concentrations of proteins from the ANGPTL family in the blood may be of great diagnostic importance in the future, particularly in the treatment and prevention of diabetes mellitus (as these proteins are involved in the metabolism of lipids and glucose), and neoplastic lesions (mainly connected with angiogenesis and metastases), especially in the course of the neoplasm, where diet, as in the case of diabetes, plays an important role (i.e., for colorectal cancer) [9,13,22,26].

## 3. Angiopoietin-like Proteins in Colorectal Cancer

The development of cancer is mainly related to environmental factors, somatic mutations, and genetic predispositions. Environmental factors include a number of physical and chemical mutagenic factors, contributing to the formation of mutations in the genetic apparatus of the cell. It has been shown that the cause of neoplastic transformation of cells are mutations in genes, mainly in proto-oncogenes, neoplastic suppression genes, and in genes whose products are involved in DNA repair. Tumor promotion and progression depends on modulator genes—the products of that are connected with angiogenesis, determine cell adhesion, and enable migration of tumor cells to blood and lymph vessels in which ANGPTL may have a part [1,6,12,13].

Angiopoietin-like proteins, apart from participating in the regulation of lipid and carbohydrate metabolism, also play an important role in the course of carcinogenesis. Their role is mainly related to the participation in many pathological processes, such as previously mentioned angiogenesis or the intensification of inflammation in the course of neoplastic changes. Colorectal cancer is one of those cancers in which the role of ANGPTL has been discovered. Therefore, we attempted to summarize the knowledge from the last 10 years regarding the participation of ANGPTL in the course of CRC. All the data described are summarized in Table 1.

### 3.1. Angiopoietin-like Protein 1

ANGPTL1 gene is downregulated in several tumor types (i.e., lung, prostate, kidney, thyroid, and bladder cancer) [32]. Chen et al. [33] revealed that ANGPTL1 was significantly downregulated in CRC samples when compared to normal tissues from The Cancer Genome Atlas (TCGA), Gene Expression Omnibus (GEO) datasets, and their own control samples. It can be suggested that ANGPTL1 is a potential suppressor of cancer and may be connected with restricted CRC initiation and development. The authors also revealed that high levels of ANGPTL1 mRNA were related to better overall survival in patients with stage IV of CRC. Moreover, after continuation of their research, the authors also revealed that higher expression of ANGPTL1 could be associated with reduction in CRC metastasis, better prognosis, and repression of CRC cell migration and invasion. In addition, the effect on proliferation and colony formation of CRC cells was limited [33]. Other researchers confirmed that ANGPTL1 expression is lower in CRC than in normal tissues, by three different methods (immunohistochemistry, western blot, and qRT-PCR assay). They also discovered that ANGPTL1 was associated with the tumor size, TNM stage, lymph node metastasis, and prognosis of CRC patients [34].

### 3.2. Angiopoietin-like Protein 2

In contrast, ANGPTL2 shows increased tissue expression and serum concentration during the course of CRC and it steadily increases with its metastatic advancement [35,36,37,38,39,40]. In addition, Yoshinaga et al. [35] reported that ANGPTL2 could be a better marker for CRC than CA 19-9, as the area under the curve (AUC) for this protein was higher than for the commonly used tumor marker. Furthermore, they revealed that a simultaneous analysis of ANGPTL2 and the C-reactive protein (CRP) serum concentrations could be useful in early detection of CRC. They also suggested that high ANGPTL2 expression may be a predictive biomarker for mucinous adenocarcinoma, as in their study, patients with this type of tumor exhibited the highest ANGPTL2 levels [35]. Furthermore, Toiyama et al. [36] discovered that serum ANGPTL2 improves preoperative detection of lymph node metastasis in CRC. In a different paper, the same authors [37] revealed that knockdown of the ANGPTL2 gene significantly inhibits cell proliferation, migration, and invasion, but enhances apoptosis in vitro, and high ANGPTL2 expression and/or serum concentration can be associated with advanced T stage, lymph node, liver metastasis, early relapse, and poor CRC prognosis. Interestingly, Horiguchi et al. [38] reported that ANGPTL2 promotes CRC cell survival after anti-neoplastic drug treatment by regulating anti-apoptotic BCL-2 family genes. They have also shown that CRC cells, which express high levels of ANGPTL2, may develop resistance to chemotherapy; therefore, attenuating ANGPTL2 signaling in tumor cells during treatment should be considered [38]. Additionally, Drebert et al. [41], based on an experiment that used human CT5.3 hTERT colon cancer-derived myofibroblasts, reported that glucocorticoid treatment could reduce ANGPTL2 concentrations, resulting in a reduced ability to stimulate endothelial cell migration and angiogenesis. Consequently, anti-ANGPTL2 therapy should be considered useful in treating CRC.

### 3.3. Angiopoietin-like Protein 4

Shantha Kumara et al. [42] revealed that ANGPTL4 plasma concentration was lower in CRC than in benign colorectal disease patients. Interestingly, the authors also reported that after minimally invasive colorectal resection, the concentrations of ANGPTL4 were even lower than before surgery. In contrary, high tissue expression of ANGPTL4 was found by Akishima-Fukasawa et al. [43] in CRC patients and was associated with lymph node metastasis and a histopathological type of CRC. Research by Zhou et al. [44] confirm that elevated ANGPTL4 expression can be found during the course of CRC and increases with the advancement of the tumor. Interestingly, their work also reveals that elevated ANGPTL4 expression could be a marker for advanced CRC. This is connected with induction of murine T regulatory lymphocytes and M2 macrophages, which could be one of the mechanisms of tumor promotion mediated by ANGPTL4. Moreover, paper by Li et al. [45] also confirmed that expression of ANGPTL4 is increased in CRC tissues. In their opinion, liver metastasis can be associated with higher expression of ANGPTL4, which enhance cell migration, invasion, and inhibit apoptosis. Similarly, Huang et al. [46] also observed higher expression of ANGPTL4 in CRC tissues and cell lines, and concluded (similarly to previous authors) that its overexpression may promote invasion and metastasis. Different researchers have revealed that the ANGPTL4 gene is upregulated in hypoxic tissues [47]. Interestingly, work by Kim et al. [48] confirms that hypoxia (induced by prostaglandin E2 (PGE2)) is connected with high expression at the mRNA and protein levels of ANGPTL4 and that this protein promotes cell proliferation and tumor growth. A paper by Alex et al. [49] demonstrates a novel mechanism of gene regulation by short-chain fatty acids (SCFA) in human T84 and HT29 colon adenocarcinoma cell lines. The transcription and secretion of ANGPTL4 is extremely stimulated by physiological concentrations of SCFA, as they play a role in activation of the nuclear receptor–peroxisome proliferator activated receptor ɤ (PPARɤ) responsible for ANGPTL4 synthesis in human colon adenocarcinoma cells. Moreover, Bai et al. [50] checked if ANGPTL4 could be a useful marker for recognizing patients who would more likely benefit from bevacizumab therapy. They concluded that the addition of bevacizumab to chemotherapy revealed patients with lower ANGPTL4 concentrations. In a paper by Shen et al. [51], the authors checked whether knockdown of ANGPTL4 and NADPH oxidase 4 (NOX4) would provide new targets to improve the outcomes in patients with hyperlipidemia-associated CRC metastasis. They revealed that knockdown of ANGPTL4, NOX 4, and other parameters dramatically inhibited circulating tumor cell extravasation and metastatic seeding of tumor cells in the lungs, indicating that the ANGPTL4/NOX 4 axis is critical for dyslipidemia-associated tumor metastasis.

### 3.4. Angiopoietin-like Protein 5

Little is known about ANGPTL5 in CRC, but Chen et al. [33] revealed that this protein was significantly downregulated in CRC samples.

### 3.5. Angiopoietin-like Protein 6

Marchio et al. [52] revealed that the interaction between hepatic ANGPTL6 and tumoral complex of α-6-integrin and E-cadherin leads to liver homing and colonization by cancer cells. Moreover, dysregulation of ANGPTL6 and its receptor interaction inhibits liver metastasis. Interestingly, co-expression of α-6-integrin and E-cadherin in primary cancers represent a poor prognostic factor for patients with advanced CRC. Additionally, the ANGPTL6-mimicking peptide has the ability to interfere with this interaction, having an anti-metastatic effect, which may be useful in anti-tumor therapy.

### 3.6. Angiopoietin-like Protein 7

Chen et al. [33] revealed that ANGPTL7 was significantly downregulated in CRC samples. Contrarily, Parri et al. [53] and Lim et al. [54] revealed that it is overexpressed in CRC tissues. According to Parri et al. [53], expression of ANGPTL7 is upregulated under hypoxic conditions. Additionally, ANGPTL7 is able to promote proliferation, migration, invasiveness, and tube formation of endothelial cells, as well as induce vascularization. On the other hand, Lim et al. [54] suggest that ANGPTL7 can have anti-angiogenic properties, as it significantly reduces liver metastasis formation and angiogenesis. They also discovered that myeloid cells (which are known to mediate metastatic progression) promote liver metastasis by downregulation of ANGPTL7 expression. The effect of ANGPTL7 is still undefined, due to the observed differences between the studies performed.

## 4. Summary

Angiopoietin-like proteins certainly play an important role in colorectal cancer development. Some of these proteins, in the course of colorectal cancer, show an increase or a decrease in concentration. For example, based on the cited studies, it can be concluded that, during the development of this type of cancer, the balance between the concentrations of ANGPTLs 1 and 2 are disturbed [33,35,36,37,38,39,40]. There is a significant reduction in the expression of ANGPTL1, which has anti-angiogenic properties, in favor of ANGPTL2, with pro-angiogenic properties, due to which, there is an enhanced angiogenesis supporting the development of cancer and its metastasis [33,35]. Based on the published data, it can also be assumed that the increase in expression and, thus, the concentration of ANGPTLs with anti-angiogenic properties, could also be important during anti-cancer therapy. This has not been investigated to date; however, it was shown that ANGPTL1 might be associated with restricted CRC initiation and development, as well as better overall survival (especially in patients with advanced CRC), better prognosis; and repression of colorectal cancer cell migration and invasion, which clearly reduce metastasis [33]. Interestingly, in the case of ANGPTLs 2 and 4, the opposite relation was shown in the case of metastases. In both cases, while high concentrations or expression of ANGPTLs 2 or 4 were observed, they were associated with both lymph node and distant metastases. This information may also be useful in anti-cancer therapy.

In addition, high concentrations of ANGPTL2 were associated with early relapse and poor CRC prognosis [37]. In the case of some angiopoietin-like proteins, apart from their pro- or anti-angiogenic properties, their concentrations or expressions were also dependent on the stages of the neoplastic changes. In the case of ANGPTL1, it was shown that, with the advancement of a tumor, the concentration of the tested protein increases. Moreover, in the case of ANGPTLs 2 and 4, an inverse relationship was observed [34,37,44]. Interestingly, under hypoxic conditions, ANGPTL4 mRNA expression and protein level may be increased, which may promote tumor invasion, proliferation, growth, and metastasis [47,48]. This may indicate its usefulness in determining the stage of neoplastic lesions, which was confirmed by other authors [44]. This information may be useful when searching for new biomarkers for CRC, as the currently used markers (CA 19-9 and CEA), due to their low diagnostic sensitivity and specificity, are not used in routine laboratory diagnostics. This path was started by Yoshinaga et al. [35], who showed that ANGPTL2 is a better marker for CRC than CA 19-9, and in combination with CRP, is highly useful in detecting early CRC changes. We believe that the continuation and extension of the research described above, with new proteins from the ANGPTL family, could be extremely useful in the diagnosis of CRC.

**Figure 3:**
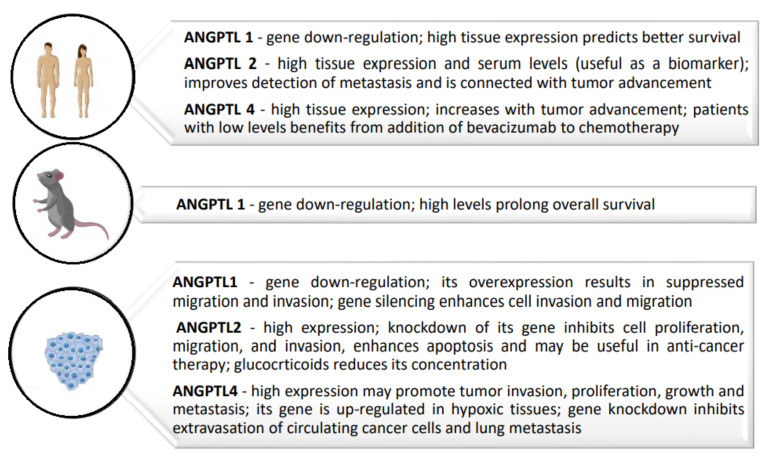
Summary of the most important data concerning angiopoietin-like proteins in colorectal cancer divided into human, mouse, and cell culture studies.

Most importantly, some of the ANGPTLs were shown to be useful in anti-cancer therapy. Studies on ANGPTL2 have shown that it can contribute to colorectal cancer cell proliferation, migration, and invasion, promote CRC cell survival after anti-neoplastic drug treatment (by regulating anti-apoptotic BCL-2 family genes), and develop resistance to chemotherapy. However, glucocorticoid treatment can reduce ANGPTL2 concentrations, which is why an anti-ANGPTL2 therapy should be considered useful in treatment of CRC [37,38,41]. In the case of ANGPTL4, knockdown of its gene radically inhibited the extravasation of circulating cancer cells and the metastasis of cancer cells to the lungs, which may prove useful in therapy [51]. On the other hand, in the case of ANGPTL6, it was demonstrated that the ANGPTL6-mimicking peptide exhibits anti-metastatic properties, which may turn out to be extremely important, especially for patients with liver metastasis [52]. The concentrations and initial work on the participation of selected angiopoietin-like proteins in anti-cancer therapy are justified and should continue. Currently, the greatest interest is related to ANGPTLs 2 and 4, while work on other members of the ANGPTL family should be continued, not only in the field of anti-cancer therapy, but also as new tumor biomarkers, which could be used in the early diagnosis of CRC. The summaries of the most important data concerning the ANGPTL family in CRC, divided into human, mouse, and cell culture studies, are shown in Figure 3.

## 5. Conclusions

In conclusion, a review of the available literature indicates that angiopoietin-like proteins play an important role in the pathogenesis of colorectal cancer development. ANGPTL1 has anti-angiogenic properties and inhibits the development of CRC, while ANGPTLs 2, 4, and 6 show pro-angiogenic hallmarks. Moreover, some of the factors were dependent on the stage of the tumor (ANGPTLs 1, 2, and 4), its type (ANGPTLs 2 and 4), the presence of metastases (ANGPTLs 1 and 4), or hypoxia (ANGPTLs 4 and 7). Importantly, some of the angiopoietin-like proteins, such as ANGPTLs 2, 4, and 6, show usefulness in anti-cancer therapy. Therefore, we suggest that investigation on the utility of angiopoietin-like proteins should be continued, especially as potential markers of CRC in the diagnosis and treatment of this type of cancer.

## 6. Literature Search and Data Extraction

**Figure 4:**
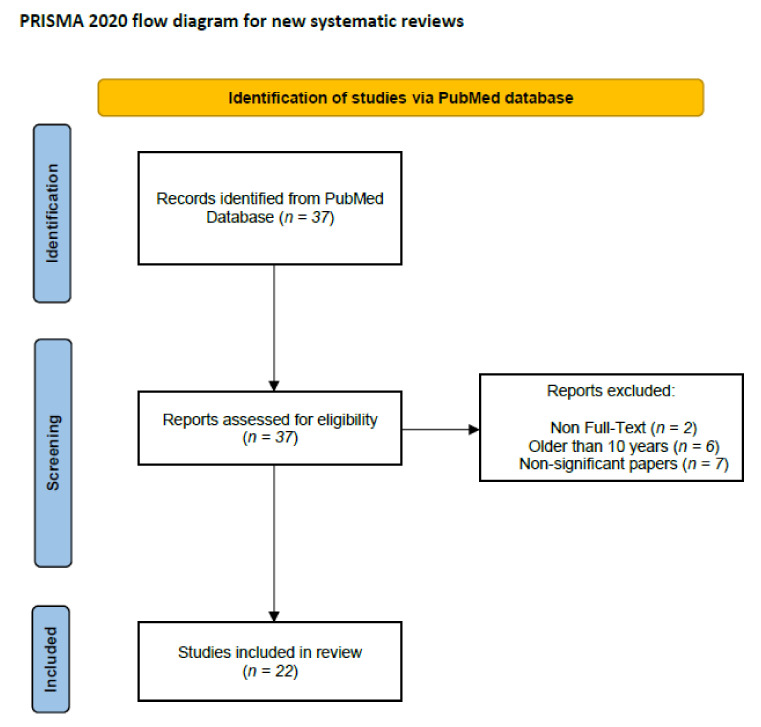
Schematic illustration of articles included in the review.

We performed a comprehensive literature search using the MEDLINE/PubMed electronic database on 20 May, 2021, with the following search strategy: ‘(Colorectal Cancer) AND (Angiopoietin-like Protein)’. For first data search, we found 37 papers, respectively. We also applied two additional filters: full-text in text availability and 10 years for publication date. In regard to the data search exclusions, we found 29 papers. In the next step, we excluded all non-significant papers (i.e., papers that did not concern angiopoietin-like proteins, colorectal cancer, or papers without necessary data). Finally, 22 publications were included in the study. All steps are included in the PRISMA 2020 Flow Diagram (Figure 4) [55]. 

## Figures and Tables

**Table 1:** Involvement of angiopoietin-like proteins in colorectal cancer.

Angiopoietin-like Protein		1	2	4	5	6	7
Concentration/Expression		↓	↑	↑	↓	↑	↑
Dependence On:	Cancer Stage	+	+	+			
	Tumor Type		+	+			
	Hypoxia			+			+
	Angiogenesis	+	+				+
	Metastasis	+	+	+		+	
Useful in Therapy			+	+		+	

## Data Availability

Not applicable.
